# ‘I think that's what I heard? I'm not sure’: Speech and language therapists’ views of, and practices in, phonetic transcription

**DOI:** 10.1111/1460-6984.12740

**Published:** 2022-06-17

**Authors:** Sarah White, Anne Hurren, Sarah James, Rachael‐Anne Knight

**Affiliations:** ^1^ Leeds Beckett University Leeds UK; ^2^ City, University of London London UK

**Keywords:** confidence, CPD, phonetic transcription, practices, speech and language therapy

## Abstract

**Background:**

Phonetic transcription is recognized in regulatory standards as an essential skill for Speech and Language Therapists (SLTs) in the assessment, diagnosis and management of clients with speech difficulties. Previous research has identified that approaches to phonetic transcription vary, and that SLTs often lack confidence in transcribing. However, SLTs’ views and working practices have not been investigated in detail, particularly in terms of whole service approaches and following the recent increase in telehealth.

**Aims:**

To investigate SLTs’ views about phonetic transcription, their working practices at both individual and service levels, and the factors that influence these.

**Methods & Procedures:**

A total of 19 SLTs from the UK were recruited to online focus groups via social media and local networks. Participants discussed their views of, and practices in, phonetic transcription. Themes were identified using reflexive thematic analysis.

**Outcomes & Results:**

Three broad themes were generated division and unity; one small part of a big job; and fit for purpose. SLTs were uniformly proud of their ability to phonetically transcribe and viewed this as a unique skill, but clear differences existed between different groups of SLTs in their views and practices. Investing in phonetic transcription was not always a priority for SLTs or services, and although many felt under‐confident in their skills they considered these to be adequate for the populations they usually encounter. SLTs make an early judgement about possible therapy targets, which influences the level of detail used in their phonetic transcription. Practical barriers are often not addressed at service level, and assessment via telehealth poses some specific challenges.

**Conclusions & Implications:**

SLTs and services would benefit from increased investment in phonetic transcription in terms of time, opportunities for continuing professional development (CPD) and initiatives such as electronic patient records (EPRs) which support the use of phonetic symbols. Identifying target sounds at an early stage raises questions about the implications of disregarding other features of speech, and the selection of appropriate intervention approaches. Further research is needed to analyse actual rather than reported practices, and to consider the relationship between phonetic transcription and intervention approaches. Future studies could also identify precise CPD requirements and evaluate the effectiveness of CPD.

**What this paper adds:**

**What is already known on the subject**

- Previous research has demonstrated that SLTs often lack confidence in phonetic transcription and that practices are varied, with relatively little use of narrow transcription. SLTs are interested in opportunities to maintain and develop transcription skills but do not often undertake CPD for transcription.

**What this paper adds to existing knowledge**

- By using focus groups as a forum for discussions, this study provides a rich and detailed insight into SLTs’ views about clinical transcription and their working practices, with previously unreported details about the reasons for these practices in a clinical context and at a service‐wide level.

**What are the potential or actual clinical implications of this work?**

- Transcription is often de‐prioritized in non‐specialist contexts, with practical barriers and a lack of clear and consistent protocols at a whole‐service level. There is an opportunity for service managers to address the systemic difficulties in using transcription effectively by raising the profile and value of transcription amongst clinicians, and promoting CPD opportunities, using the findings of this study as a rationale for funding this. Together, these recommendations have the potential to improve client outcomes through more accurate assessment and diagnosis, and hence more appropriate intervention.

## INTRODUCTION

Phonetic transcription using the International Phonetic Alphabet (IPA, 1999) is an essential component in the training of all Speech and Language Therapists (SLTs), and is included in the Health and Care Professionals Council's Standards of Proficiency for SLTs (HCPC, 2014). The Child Speech Disorder Research Network notes that ‘transcription ability is a skill unique to SLTs […] no‐one else can provide this information about a child's speech’ (CSDRN, 2017: 2). Thus, SLTs are unique amongst healthcare and primary/secondary education professionals in their ability to transcribe speech phonetically, and indeed to listen to speech objectively.

There are numerous approaches and levels of detail that can be employed in transcription: Heselwood (2013) lists several (albeit not mutually exclusive) dichotomies such as specific versus generic, speaker versus listener orientated, and systematic versus impressionistic, along with other categories such as phonemic, allophonic and segmental transcription. A key distinction is the broad–narrow continuum, which ranges from narrow phonetic transcription capturing specific details of physical utterances to broad phonetic transcription capturing physical utterances in less detail, through to phonemic transcription which captures only the contrastive categories used rather than the specific realizations. One approach is not inherently superior to any other; rather, Heselwood (2013: 25) proposes that the quality of a transcription may be judged by how well it fulfils its intended purpose.

In clinical practice, phonetic transcription and subsequent analysis is used to inform differential diagnoses of particular categories of speech sound disorder (SSD), which have different aetiologies, for example those which are phonological in nature compared with those which are secondary to organic causes such as cleft palate or hearing impairment (e.g., Dodd, 2014). Transcription is also used to select clinical targets and intervention approaches, and to provide a baseline against which progress can be monitored (CSDRN, 2017). Recommendations for transcription are provided in the Good Practice Guidelines for the Transcription of Child Speech (CSDRN, 2017)—there is no equivalent guidance for adult speech, although many of the recommendations are applicable to both groups. The level of detail employed is at the discretion of the individual clinician: the guidelines note that SLTs may use either broad or narrow transcription as needed; a position which supports Heselwood's (2013) view, described above.

In a review of 320 SSD referrals over a 15‐month period, over 87.5% were identified as phonological rather than articulatory in nature (Broomfield & Dodd, 2004). The practice of using broad transcription for phonological errors is endorsed by the CSDRN (2017: 8), and in a survey of SLTs in the UK (Knight et al., 2018: 780) 40.6% reported using only broad transcription with an exemplar rationale being, ‘I use broad transcription as I find it meets my needs’. For speech errors that are not found in typical development, Howard and Heselwood (2002: 373) believe this ‘will clearly require transcription at a phonetic, rather than phonemic, level’, implying that atypical speech by definition cannot be adequately captured by a phonemic transcription. This is not necessarily the case, however: some subtypes of SSD such as consistent phonological disorder and inconsistent phonological disorder are characterized by errors which, although atypical, are phonological rather than articulatory, such as initial consonant deletion, or inconsistent phonological substitutions (Dodd, 2014). Conversely, typical speech errors (either age appropriate or late‐persisting) may require more detailed transcription for errors such as feature synthesis in consonant clusters such as [n̥] for /sn/ (e.g., Chin & Dinnsen, 1992), suggesting a false dichotomy between the practice of using broad transcription for typical errors versus narrow transcription for atypical errors.

For clients with atypical articulation (as opposed to atypical phonological) errors, much of the literature advocates the use of narrow transcription, including use of the ExtIPA symbols for disordered speech (IPA, 1999). Muller and Papakyritsis (2011) and Ball et al. (2009) present examples of clinically significant features captured by a narrow transcription, demonstrating how transcription can inform the subsequent intervention. Narrow transcription is recommended for cleft palate or hearing‐impaired speech in clinical guidance (CSDRN, 2017), and was reportedly used by specialist SLTs working with those populations in Knight et al.’s (2018) survey. Studies involving SLT students have similarly found that their experience of seeing or using narrow transcription is almost exclusively restricted to cleft palate and hearing impairment contexts (e.g., Shaw & Yanushevskaya, 2021; Windsor, 2011). When recommending narrow transcription for these populations, however, the CSDRN guidelines do not specify whether all segments should be transcribed narrowly (including, for example, acceptable allophonic realizations) or only the disordered realizations, and in Knight et al. (2018) those who used narrow transcription did not specify whether this applied to all segments. As an alternative to narrow transcription, Meyer and Munson (2021) propose using a perceptual rating scale, for example, on a spectrum between a ‘[t]‐like sound’ and a ‘[k]‐like sound’, while Roxburgh et al. (2016) found that SLTs’ perceptual evaluations (without using traditional transcription) were adequate for assessing the speech of children with repaired sub‐mucous cleft palate.

Thus, the question remains about whether (and how) clinicians might make judgements about which aspects of speech require more detailed transcription and in which situations they should transcribe more tokens to facilitate analysis. Howard and Heselwood (2002) warn that clinicians should avoid judging clinical relevance too early, and with reference to ophthalmology, Hussain and Oestreicher (2018: 120) describe a number of cognitive biases in clinical decision‐making and diagnosis, whereby ‘a failure of heuristics may lead to diagnostic error’. An early judgement of clinical relevance may therefore cause the listener to miss details which may not be immediately salient, yet have implications for the diagnosis and subsequent therapy input for clients. Different SSDs require specific intervention approaches; for example a phonological intervention such as Multiple Oppositions Therapy would be inappropriate for children with non‐phonological SSDs such as childhood apraxia of speech. Accurate diagnosis, which is informed by accurate transcription, therefore holds significant implications for the efficacy of clinical interventions, as well as considerations relating to time‐ and cost‐effectiveness.

Further to its application in speech assessment and diagnosis, transcription is also used to monitor progress (CSDRN, 2017: 8) and to evidence therapy outcomes (e.g., Enderby et al., 2009). Routinely incorporating transcription into reassessment—a common practice reported by SLTs in Knight et al.’s (2018) survey—could demonstrate progress between two points in time (pre‐ and post‐intervention) and, in a broader context, demonstrate the value and effectiveness of SLT interventions service to the commissioning body (Children's Commissioner, 2019: 7). However, in a review of 174 sets of clinical case notes, Morgan et al. (2021) found that SLTs did not consistently record or analyse pre‐ and post‐intervention data in sufficient detail to adequately monitor progress and to evidence outcomes for children with SSD.

Research describing the clinical use of transcription has primarily focused on organic SSDs such as cleft palate speech (e.g., Roxburgh et al., 2016; Sell, 2004) or hearing‐impaired speech (e.g., Teoh & Chin, 2009). Much less has been published about transcription (and subsequent analysis and decisions about clinical pathways) for other client groups, such as children with other types of SSD, or adult clients. In a survey of 333 SLPs, Skahan et al. (2007) report that many favoured particular published screening assessments and were over‐reliant on these tools to make decisions about clinical pathways for children, without sufficient analysis of speech alongside the screening assessment. A survey of 231 Australian paediatric speech–language pathologists (SLPs) found that typical assessment consisted of single‐word sampling, stimulability testing and judgement of intelligibility (Baker & McLeod 2014). The study did not investigate transcription specifically, although 85.6% reported that their assessment ‘sometimes’ or ‘always’ included consideration of the client's phonetic inventory. Joffe and Pring (2008) found that the majority of 98 participating SLTs rated themselves as ‘confident’ or ‘very confident’ in selecting SSD interventions, but again did not investigate the role of transcription in this decision‐making process. In a survey of SLTs working with a range of client groups, Knight et al. (2018) found that many lacked confidence in using transcription, particularly narrow transcription. This cohort also reported limited opportunities to maintain skills after pre‐registration training, although 75% expressed an interest in opportunities for continuing professional development (CPD). Both Windsor (2011) and Knight et al. (2018) hypothesize a ‘theory to practice’ gap in SLTs’ use of transcription, suggesting a difference between the advanced level at which transcription is taught to SLT students and the more basic level which is subsequently used in clinical practice. In the context of limited guidance from professional bodies about the transcription skills needed upon qualification, Titterington and Bates (2021) make a case for developing benchmark competencies at various levels, from newly qualified practitioner (NQP) to generalist and finally specialist SLTs (e.g., those working with cleft palate and hearing impairment caseloads), in order to support the maintenance and development of transcription skills in clinical practice.

Practical barriers to transcription, such as limited time for detailed transcription or for CPD, have been highlighted previously (Titterington & Bates, 2021; Windsor, 2011), and recently a further challenge has arisen following the increase in the use of telehealth. During the COVID‐19 pandemic, a survey by the Royal College of Speech and Language Therapists (RCSLT, 2020) found that 63.1% of SLT respondents were using online platforms for client‐facing sessions. The inherent challenges of telehealth models—including connectivity, technical difficulties and distortions of the acoustic signal—have been noted by Sevitz et al. (2021) and Campbell and Goldstein (2022), but neither study considers transcription specifically. Little is known about SLTs’ ability to transcribe effectively via telehealth, and the RCSLT (2020) identified a need for further research into the use of telehealth with particular client groups (although did not specify which groups).

To date, there is little in‐depth qualitative research into SLTs’ practices and views about transcription, particularly amongst generalist SLTs (those whose caseloads are mixed rather than solely SSD clients). Knight et al.’s (2018) online survey provides a useful insight into the views and practices of SLTs working with a range of client groups: while this study design enabled sampling of a large number of SLTs (*n* = 759) it could not facilitate opportunities for in‐depth discussions or probing clinicians’ views and practices in detail. A further gap in the literature concerns service‐wide considerations such as explicitly adopting a consistent approach to transcription, implementing and promoting existing professional guidance, or service‐wide barriers and facilitators to transcription. The shift towards telehealth also requires studies to establish whether and how clinicians’ views of and practices in transcription have changed.

The present study seeks to provide further in‐depth information about these areas through the following research questions:

What are SLTs’ views about clinical phonetic transcription?

What are the working practices of SLTs and SLT services in relation to transcription, and what factors influence these practices?

## METHOD

### Background

Ethical approval was granted by Leeds Beckett University; the participants gave written, informed consent to take part in the study. The first author, who conducted this research, is a qualified SLT and a university lecturer in SLT, with 14 years’ clinical experience (primarily with children with SSD).

### Participants

Inclusion criteria were: (1) currently working as a SLT in the UK; and (2) working with clients with speech difficulties for at least part of their time. Participants were not required to use transcription in their role, as it was deemed important to include SLTs who may not transcribe and to explore the reasons for this. A total of 19 participants were recruited using purposive sampling via social media and existing local networks of SLTs: one of these networks was a regional CPD transcription group run by the first author.

### Materials and procedure

Participants each attended one of five online focus groups, which were held out with participants’ working hours via Microsoft Teams and video recorded. Groups were organized for convenience rather than by specialism, and group size ranged from three to five participants.

The focus group schedule (see Appendix 1 in the additional supporting information) was piloted by the first author with two SLTs, and minor changes to the questions were made as a result. Questions were designed to act as prompts for discussion (Braun & Clarke, 2013), covering topics such as working practices and approaches to transcription (at both individual and whole‐service levels), CPD experiences, confidence, perceived barriers to transcription and the clinical application of transcription. Participants were also asked to describe a recent experience of transcribing a client's speech. Focus groups lasted between 66 and 85 min (average = 73 min). The videos were orthographically transcribed verbatim, with ‘fillers’ (e.g., *um, well*) removed in the excerpts quoted. Details about participants are presented in Table 1.

**TABLE 1:** Participant information

Participant information	*n* (%)
*Recruitment*	
Local networks	12 (63.2)
Twitter	7 (36.8)
*Relationships*	
Known to the moderator (through CPD group or otherwise)	10 (52.6)
Not known to the moderator	9 (47.4)
Attended the moderator's CPD group	9 (47.4)
Never attended the moderator's CPD group	10 (52.6)
*Clinical background*	
General paediatric	13 (68.4)
Cleft palate specialist	2 (10.6)
Hearing impairment specialist	1 (5.3)
Speech sound disorder specialist	1 (5.3)
Mixed (cleft palate/general paediatric)	1 (5.3)
Apraxia of speech (adult)	1 (5.3)
*Experience*	
Autonomous practitioner	17 (89.5)
Newly qualified practitioner	2 (10.6)
*Employer*	
NHS Trusts (*n* = 9)	14 (73.7)
Social enterprise (*n* = 1)	2 (10.6)
Independent practitioner	1 (5.3)
Employed by school	1 (5.3)
Not currently employed	1 (5.3)

### Analysis

Focus group data were analysed using the six stages of reflexive thematic analysis described by Braun and Clarke (2006, 2019). Familiarization with the data was achieved through initial orthographic transcription of the videos and subsequent re‐readings of transcripts and rewatching of videos, both before and during the coding stage. Open codes were generated through giving equal attention to each item in the data and applying labels such as ‘pride in specialist skill’ and ‘red tape and technology’. Many codes were refined, split or merged during two further coding sweeps, and 26 final codes were generated. To identify broad patterns across the data, codes were grouped together for similarity, and early theme and sub‐theme candidates created: some had been codes in their own right while others were generated from groups of similar codes. Themes and sub‐themes were then reviewed, refined and named.

## RESULTS

**FIGURE 1:**
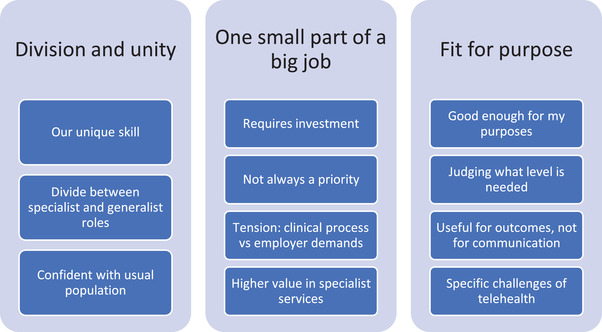
Themes and sub‐themes [Colour figure can be viewed at wileyonlinelibrary.com]

Three broad themes were generated from the analysis: (1) division and unity; (2) one small part of a big job; and (3) fit for purpose. Figure 1 shows the themes and sub‐themes.

### Theme 1: division and unity

SLTs identified that transcription is essential to the work of SLTs, with 10 participants stating, unprompted, that it was vital for their role, for example: ‘I wouldn't be able to do my job, I don't think, without transcription.’ One SLT who worked with a general paediatric caseload stated, ‘it's rare that a day would go by when I don't transcribe’. There was also a recognition that transcription is unique to the SLT profession within healthcare more broadly, and many expressed pride in this unique skill:

It is definitely something that's so unique to us and we sort of take it for granted cos even when you find it difficult […] we've got at least some knowledge of it and that is a very unique skill.

Some also discussed feeling excitement and pride in the process of learning to transcribe as a student: ‘look, I can do funny symbols […] I felt like a bit of superiority’. Thus, SLTs felt that transcription was an inherent part of their identity, and that this skill both unites them and also distinguishes them from other healthcare professionals.

However, SLTs working with general paediatric caseloads described a clear divide between their own role and that of specialist SLTs such as SSD, cleft palate or hearing impairment specialists. SLTs appeared to identify both themselves and their service as either specialized or not specialized in using transcription, indicating that despite their view of transcription as a specialist skill for the profession, they have different perceptions about their skills in using transcription and its application to their own role. Non‐specialist (generalist) SLTs frequently discussed ‘the specialist’, framing the specialist as an inherently different type of SLT, with the onward referral marking a clear boundary between the two roles. Onward referrals were made when it became apparent that more in‐depth transcription and more specialist management was required, for example, for children with multiple atypical speech errors:

If I feel like narrow transcription is needed, I try, I try my hardest but usually I'm like ‘OK and now I'm gonna pass you on to our specialist’ because it's a child that, they're gonna be too complicated for me.

If a child comes in the door and the first thing that it says is a bunch of vowels, you're instantly going [*sitting up and widening eyes*]—cos you know you're going to transfer them, you know you're getting a specialist involved [*laughing*].

Similarly, those who identified as specialists in transcription also perceived inherent differences between themselves and generalist SLTs: one cleft palate specialist described referrals from generalists where ‘they are wrong’ (although she acknowledged that ‘we'd prefer that than not getting the referral at all’).

There were also differences in the use (or not) of specific symbols used by SLTs depending on which populations they usually work with. One generalist SLT felt that repeatedly using the CLEAR phonology assessment (Keeling & Keeling, 2006)—which was consistently described by generalists as their ‘go‐to’ assessment—with similar clients had led to her becoming accustomed to completing the assessment as if by rote, using the same English phoneme symbols each time to capture typical phonological errors: ‘the majority of my transcription is the CLEAR, and it's almost now become muscle memory. And then I feel like I've lost that wider skill of being able to transcribe’. Indeed, the CLEAR assessment does not necessarily require transcription at all, and some SLTs reported using ticks for correct realizations and circles around errored sounds on the printed orthographic words: ‘we'll circle the sound as we're doing it live, so that we can quickly see what sounds they're not producing’. On the whole, generalist SLTs were confident in identifying errors such as phoneme substitution or omission through objective listening (with or without transcribing), and some were also able to identify certain sounds not typically found in English speech that they encounter frequently: ‘I think there's a few now that I know, like bilabial fricative, I find that quite a lot with kids producing an <f> as a bilabial fricative so I know that now’. However, many expressed doubts about their ability to perceive less common errors: ‘I'm constantly thinking, “I think that's what I heard? I'm not sure, I think that was it.”’ One SLT described feeling ‘consciously incompetent’ at transcription, adding ‘I always feel if something more disordered walks through the door I can't transcribe it as accurately as I want to […] I'm constantly just waiting for that person that's going to challenge that.’ Others found certain symbols difficult to remember:

I do have ones that I always forget, like […] voiced palatal fricative, I can always remember the voiceless but then I have to really think about the voiced one and I guess the same for vowels as well.

Generalist SLTs employed strategies such as annotating assessment forms to indicate and describe sounds that were difficult to transcribe:

I'd write it down as best I can and I'd put some type of mark on this sound that I know I've not transcribed to be the sound it is [*laughing*], and then […] write down something along the lines of, mouth isn't open enough, or tongue's too far back.

SLTs working with cleft palate or hearing impairment clients were more likely to use ExtIPA symbols and/or additional diacritics, and to view this as essential for their role, but again tended to describe this in terms of specific symbols to capture the features they commonly encounter within their specialist population:

A common one they, for <f>, it looks as though they're saying [f] cos they mark the place but it's still stopped, there's no fricative so it's just like [*silently demonstrating labiodental plosive*] and how to transcribe that is now a new fave of mine cos I just see it all the time, but I know how to transcribe it. (hearing impairment specialist)

I'll note down the kind of resonance features or airflow features […] I would use diacritics all the time to indicate nasality, nasal emission, nasal turbulence. We would use weakness quite a lot, by virtue of VPI, but also for dysarthric patients. (cleft palate specialist)

Similar to generalist SLTs, specialists did not necessarily feel confident about transcribing features not commonly found in the populations they usually work with: a hearing impairment specialist commented, ‘I think I'd still feel nervous if you gave me a cleft child’, and a cleft palate specialist described feeling unsure about symbols for certain vowels. Four SLTs in total described difficulties with vowel transcription, for example, ‘I rarely get a child that makes vowel errors but when I do it does throw me a little bit’—although it was unclear whether ‘errors’ referred to distortions or substitutions.

### Theme 2: One small part of a big job

SLTs identified that transcription is a skill which requires an investment of time, both for the quality of transcription in each session and also for the maintenance of one's own skills over the course of a career. The length of a session is often limited by service‐wide protocols: ‘we have to try and do the assessment and do the notes and write a report within an hour’ (one SLT reported an even shorter limit of 30 min). For SLTs with more flexibility, time was still a consideration when planning clinical activity across a caseload: ‘I can't spend too long on transcribing one particular piece of work when I know that I've got a backlog of other bits that need to be done.’ Time limitations were also described in relation to schools who directly commission SLT input:

Being able to evidence that [time] back to people who are commissioning you in school being like ‘so where were you all afternoon?’ like, ‘oh you know that one boy? Well I wanted to sit and listen to everything that he was doing’ and they look at you like, ‘are you mad?’.

The need to remain mindful of service protocols, caseload management and commissioner satisfaction suggests that there is often a tension between the desire to complete clinical processes to a high standard whilst also meeting the day‐to‐day demands of one's employer. Thus, the time required to complete a detailed transcription, or to transcribe more tokens, means that transcription is not always a priority for individual SLTs within a session.

SLTs also recognized that within a clinical session there are numerous clinical aspects to observe and record. Many did not transcribe speech as part of a language assessment, for example if clients produce phonemic paraphasias or incorrect realizations: ‘I'm more focused on their language […] speech, it just goes a bit out the window for me’—although some reported using phonemic transcription if the target word or the scoring was ambiguous:

If it's not pronounced properly I will transcribe it sometimes, because you know sometimes when they say /skɛlɪskəʊp/ [*telescope* …] I think you can get a point [on the assessment] for /skɛlɪskəʊp/ but I need to look back in the manual to see how near it was to the target word in order to get the point.

Transcribing can also prove difficult to manage whilst also engaging the client, particularly when working with young children:

At university you'd have minimal distractions [when transcribing], then in a clinic room, the child could be throwing the foam dice around the room, they could be hiding under the table, so I think in clinic there's more distractions.

Most generalist SLTs reported that they transcribe single words, in formal assessments only, rather than transcribing connected speech in assessment sessions or transcribing at any level in therapy (as opposed to assessment or reassessment) sessions:

I know I probably should transcribe during therapy sessions as well, but I must say I do that less. On top of everything else you've got to remember that's sort of a second thought for me.

One SLT summarized all of these difficulties succinctly, saying ‘it's one small part of a big job’, capturing the notion that, for many reasons, transcription is not always a priority for SLTs within clinical sessions.

Maintaining or improving transcription skills also requires investment, and many SLTs discussed their experiences of and their interest in CPD for transcription. All SLTs reported that they value reassurance from others, such as colleagues, CPD tutors or fellow CPD delegates, and reflected on the benefits of consensus transcription:

If I transcribe something similar to whoever is sat near me [in a CPD session], I'm like, ‘oh yeah, I got it right’ […] I just like knowing I've got a similar thought to everybody else around me and I think when you work by yourself so much you don't have that with anybody.

The transition from student to NQP, in terms of opportunities for reassurance, was also commented on by one NQP: ‘you had so much support at uni and then someone's trusting you to transcribe’. Generalist SLTs did not routinely attend transcription CPD sessions, with the exception of the first author's regional group (attended by nine participants, of which seven were generalist SLTs), which was free of charge and held out with working hours. SLTs made suggestions for how CPD could be delivered: one proposed ‘a phonetic transcription revision session, like an online thing, and we can log in in once a year and you could have like a revision package’, while another suggested ‘we could all bring case studies’. A hearing impairment specialist reflected that students can also provide reassurance to qualified practitioners: ‘of all the things to do together with a student, transcribing a speech assessment together is a really nice activity and a really good learning activity […] it's really helpful for us as well’, suggesting that both novice and experienced transcribers value the opportunity to calibrate one's own transcription with others.

However, the investment of time for CPD was, again, not always a priority at a whole‐service level:

We had some conversations about trying to set something up in the team where we did more joint transcribing as a CPD thing […] but it's just trying to fit it in with everything else in the service, it's quite hard.

Two SLTs in who worked in the same service discussed the difficulties they faced in asking their employer to fund CPD courses:

You'd have to link it into your appraisal and your learning objectives, and it's quite a specific thing to put down if you're not in that specialist area, so I don't know if they'd let you access it.

Supporting the notion of a divide between specialists and generalists as discussed under Theme 1, transcription appeared to hold a higher value in cleft palate and hearing impairment services, where investment in practices such as audio‐recording, longer clinical sessions and attending CPD courses was more common:

I've been working with hearing impaired children for two years now, so I've been on some courses and I've annotated my IPA chart and I have that next to me so I just spend a lot longer doing it in more detail.

Similarly, consistent whole‐team approaches to transcription were described by one cleft palate specialist:

We record all of our VPI assessments so that's where children come where there's a concern about the palate and they'll have x‐rays as well, so on those days they'll have recordings and our audit clinics are also recorded.

A lack of investment in transcription practices in generalist services was also evident in SLTs’ accounts of technical difficulties in producing and storing electronic transcriptions. Many described electronic patient record (EPR) systems which do not support IPA symbols, meaning that SLTs must write or type transcriptions and attach these documents separately. Some SLTs therefore attempt to capture clients’ realizations in the EPR using orthography, while others use descriptive labels for sounds: one acknowledged ‘that's weakened my knowledge of symbols, because I'll sometimes, especially for more obscure ones I'll just think to myself how to describe it [in the EPR] rather than get the symbol’. For SLTs who use EPRs which do allow IPA symbols, this was often limited to base symbols: ‘if you have to put extra diacritic bits in, I mean, I've no idea how you'd do that’. Generalist services had not prioritized investigations into how to facilitate the use of IPA symbols in EPRs (‘some therapist said that you could get [a function for IPA symbols] but we don't have it’), while conversely in specialist services such as cleft palate, ‘we put a huge bid forward for being able to transcribe [electronically …] it allows when I copy and paste in from the IPA’. Similarly, many services had not established clear policies to enable SLTs to make and store audio‐recordings of clients’ speech for subsequent analysis: one SLT reported that ‘we're not allowed to record in my trust, so it's always live […] I'd love to be able to record’, while another commented that ‘[there's] just red tape everywhere you look’.

### Theme 3: fit for purpose

Theme 1 (‘Division and Unity’) identified that generalist SLTs were confident in using broad transcription to capture phonological processes. Most felt this level was sufficient to identify therapy targets, and therefore judged their transcription skills to be good enough for their purposes:

I would say more than 90% of my transcription is only at a broad sort of phonemic level, rarely do I go beyond that actually. However I find that it's enough to do what I need to do with the vast majority of the children.

One SLT, who works with adults with Apraxia of Speech, reported that her colleagues generally use orthographic transcription, arguing that this is sufficient for identifying patterns of substitutions or omissions at a phonemic level and therefore for setting targets:

Because it's just the English symbols really, you probably could do that with orthographic transcription […] I think people have been getting along just fine without it to be honest and working with apraxia of speech and just using spelling.

This SLT acknowledged, however, that she did use IPA symbols to capture non‐English realizations made by bi‐ or multilingual clients: ‘he was a Tamil speaker so it was useful to get a straight‐up, what's his phonological inventory, to start off with’, and she also went on to reflect that transcription can invoke an underlying knowledge of place, manner and voice categories and therefore guide clinical decisions when setting targets:

I can see by using the IPA whether that is one sound or a type of sound, so in that way it would set goals like ok we're gonna work on bilabials first, maybe […] so it can help me categorise what type of sounds to go for.

This view was shared by another SLT who felt that transcription inherently reflected SLTs’ training and their ability to listen to speech: ‘it's not just learning a new alphabet, is it, which people might think it is […] we've had loads of training around using it’. Another reflected that ‘even just being able to listen and to pick out the sounds is a skill in itself, isn't it?’.

When planning intervention for clients, some specialist SLTs described how their transcription might suggest a particular diagnosis and therefore a specific care pathway or intervention:

At that prognostic level of deciding what route does a patient need to go down, which is you know, is applicable to our population, the transcription is sort of key to signposting them (cleft palate specialist).

However, generalist SLTs rarely discussed how their transcription might inform diagnosis, and actually suggested that the reverse might occur—that is, their early judgement about the type of SSD informs the transcription:

As soon as they start talking I guess I'd make some type of judgement going ‘oh okay you're just fronting yeah alright then’ […] whereas if they open their mouth and they sounded like there was something more disordered going on then I would tune in more and I would transcribe more.

I'd be making a judgement about how long‐term or short‐term it was and therefore how much time I would be spending on doing the transcription in the first place […] with a simple speech delay it would seem like not really worth doing loads of transcription.

Instead of using their transcription to inform their target‐setting, therefore, generalist SLTs used clinical judgement to anticipate what their targets might be, and then disregarded in their transcription the features that they judged to be less relevant: ‘I'd probably go for more clinically relevant or clinically not relevant [… if] I'm not going to set a target around that, it's not clinically relevant, it doesn't really matter’.

Many felt that transcription held limited value as a communication system between clinicians. While they acknowledged that clients are frequently transferred between SLTs, there is often a long period of time between the initial transcription and the subsequent intervention: ‘there would usually be a quite significant gap of time that had passed and so you probably want to do some reassessment anyway’. For this reason, many generalist SLTs reported that they did not usually read transcriptions completed by their colleagues. Others were mindful of colleagues’ confidence and ability in using and reading transcription, and felt that less common symbols might not be understood by others, suggesting that transcription is not always perceived as a core skill for SLTs:

Other therapists might not have an awareness of the diacritic, so it's almost trying to make sure that your transcription is readable for other therapists.

If that's for another therapist to look at, are they going to know what you've put […] if you've put some fancy whatever? I don't know.

In a discussion about service‐wide protocols and shared approaches to transcription, one SLT suggested that ‘a protocol for the team I think would make reading other people's transcriptions easier’.

A total of 11 SLTs described the value of transcription as a means of monitoring progress and evaluating therapy outcomes for children: ‘I've been doing a lot of therapy with a boy, I think he's got Developmental Verbal Dyspraxia, and I think the transcription has been key because it allows me to monitor his progress.’ Two others commented on the role of transcription in demonstrating the effectiveness of intervention approaches to commissioners:

We're trying to prove that Multiple Oppositions Therapy is effective and if [we] didn't transcribe we wouldn't have anything to statistically show that […] We can say ‘look how many speech sounds they were missing pre‐intervention, look at their inventory now’ and that's all [from the] transcription so I think it's really important.

SLTs reported many specific challenges associated with telehealth. Some reflected that typical phonemic substitutions were easy to perceive: ‘where it worked OK was if they were doing quite an obvious and quite clear process’; but others lacked confidence in their ability to perceive even common phonological substitutions via telehealth: ‘if there's a slight time lag, it really throws it off. And especially with [k] and [t] like fronting or backing, I just cannot tell’. Two SLTs discussed feeling more confident when using telehealth to reassess a child who they had previously met in person, because they had prior expectations about the client's speech production, although one reflected that this in itself caused some bias:

They could have totally changed their errors and you're thinking last time we saw him, ‘oh yeah, you could never get a [k], so yeah, that sounds right’. And actually they have. You just have this thought of what they're going to sound like when you go into the call.

SLTs working with all client groups felt less confident about telehealth transcription compared to live transcription, with three specialist therapists (cleft palate and hearing impairment) noting particular features that were hard to perceive:

We'd realise that mostly it was airflow errors that we weren't picking up on, and active nasal fricatives, so the fricative sounds were the ones that we were the most vulnerable on, just because of the audio link (cleft palate specialist).

One hearing impairment specialist concluded that ‘I can't do speech assessment over telehealth […] I really can't reliably transcribe’, suggesting that transcription via telehealth is not always fit for purpose, particularly with certain populations.

## DISCUSSION

This study investigated the views and working practices of 19 SLTs in relation to phonetic transcription, and the factors that influence these. Three themes were generated: (1) division and unity; (2) one small part of a big job; and (3) fit for purpose. Here, these themes are summarized and discussed, and their collective implications considered.

SLTs in this study recognized transcription as a specialist skill which they felt proud of, supporting the CSDRN's (2017) position that transcription is unique, within healthcare, to SLT. While almost all SLTs working with speech saw transcription as an inherent part of their role, clear differences in views and working practices exist between generalist and specialist SLTs. The limited use of narrow transcription by generalist SLTs in this study (and their lack of confidence in using it) is consistent with previous research (e.g., Knight et al., 2018; Windsor, 2011); however, some had evidently retained the ability to perceive sub‐phonemic details even when they could not recall or did not know the symbol, using annotations and descriptions instead. In cases where SLTs’ transcription skills were not adequate for capturing details, their management (such as making onward referrals to specialist services) was still appropriate, suggesting that SLTs’ decision‐making, if not the actual transcription, is nonetheless ‘fit for purpose’.

Many SLTs made early judgements about clients’ overall intelligibility, their ‘typicalness’ for the clinical population and their prognosis in order to choose which features to capture in transcription. SLTs often had prior expectations about what they might hear and what their focus should be, both in transcribing and in setting targets: while some discussed the role of bias specifically in telehealth reassessment, this could also apply to assessment more broadly, regardless of method. The practice of making an early judgement, therefore, must still be regarded with caution: Hussain and Oestreicher (2018: 120) note that heuristics are ‘useful in assimilating a large amount of information and distillating this into salient points’, but warn that time and workload pressures, along with being less familiar with the presenting problem, can cause bias in clinical decision‐making. This observation is particularly relevant in light of SLTs’ views that transcription is ‘one small part of a big job’ and their descriptions of strict time limits for clinical sessions.

Existing recommendations for transcription (e.g., CSDRN, 2017) recognize that SLTs may employ different levels of detail in transcription: this is borne out by reported clinical practices in this study and supports Heselwood's (2013) view that a transcription is detailed enough if it fulfils its intended purpose. However, the practices reported in this study suggest that details are often overlooked and existing clinical guidance for transcription is not followed—indeed, transcription is not always used, with SLTs sometimes using orthographic transcription or a ‘tick‐box’ system. This has significant implications for accurate analysis and diagnosis, including specific considerations relating to bi‐ and multilingual clients for whom SLTs may need to capture realizations not typically found in an English phonetic inventory. Analysis of speech without adequate transcription (either due to lack of detail or limited tokens transcribed) also has the potential to affect the selection of evidence‐based interventions for specific types of SSD, and the ability to monitor and evidence outcomes for children (e.g., Morgan et al., 2021), as well as increased likelihood of re‐referral of clients if an unsuitable intervention is chosen. While listening to speech and transcribing at any level is a skill in itself, if narrow transcription is viewed as the exclusive domain of specialist SLTs then generalist therapists risk missing potentially significant details, and being unable to transcribe and, through analysis, identify more unusual errors when needed. SLTs cannot monitor clients’ progress without transcribing, particularly as most SLTs in this study did not transcribe routinely in therapy sessions. There is a further danger, in exclusively using broad transcription and relying on one favoured assessment tool, that SLTs risk diminishing the specialist skill which they are manifestly proud of and which delineates their role from that of other professionals.

Participants in this study who had attended CPD sessions valued the opportunity to learn from others and to gain reassurance about their ability to transcribe—similarly, Knight et al. (2018) reported that peer support was suggested by many SLTs as a CPD activity. However, many SLTs rarely, if ever, attend any CPD for transcription (Knight et al., 2018), and the current study provides insight into some of the reasons for this: some generalist services are unlikely to fund CPD for transcription, which both reinforces the notion of the divide between generalist and specialist, and indicates that transcription may be viewed as a skill for which only specialist SLTs need to undertake CPD. Theme two identified that transcription is seen as ‘one small part of a big job’, and therefore CPD is not prioritized by services or individual SLTs. With few opportunities for post‐qualification CPD in transcription, SLTs may lack the skills needed to progress to specialist posts, or to succeed in such posts. The views and practices reported in this study support the case, made by Titterington and Bates (2021), for developing benchmark competencies for transcription skills at various levels, whereby targeted CPD could be undertaken to work towards each level.

These data also highlight the barriers presented by technology, including the use of adequate EPR systems and clear policies around recording clients’ speech, as well as the challenges of telehealth which has recently become significantly more widespread. These challenges include identifying nasality and place of articulation, which impacts on SLTs’ confidence when transcribing via telehealth. As this method of assessment appears likely to continue (RCSLT, 2020), the results from this study raise tentative questions about transcribing via telehealth and about whether, in the case of phonetic distortions particularly, alternatives such as a continuous rating scale (e.g., Meyer & Munson 2021) may be beneficial.

Finally, the results reported here represent wider considerations within the profession. Concerns about lack of time and funding for CPD, conflicting demands and the need to be accountable to service commissioners are not specific to the issue of transcription, and the importance of promoting the value of SLT‐specific skills could also be applied to other practices (e.g., grammatical analysis) as well as transcription. Similarly, overreliance on screening tools (e.g., Skahan et al., 2007), the use of heuristics, and the possibility of cognitive bias, might also be applied to all SLTs’ decision‐making, and indeed that of other healthcare professionals. Thus, transcription is ‘one small part’ but also reflects the ‘big job’ more broadly.

### Limitations

Although questions were used as a basis for broader discussion, some responses were prompted by key words in the questions such as ‘confidence’ and ‘barriers’. Prior relationships between researchers and participants also influenced the discussions—for example, some participants were aware of the moderator's views about transcription, which may have prompted comments such as ‘I know I probably should transcribe more in therapy sessions’. However, prior relationships were primarily a supportive factor in establishing rapport.

Participants in this study were, by definition, likely to have an interest in transcription (indeed some explicitly stated this) and to use transcription routinely. Adult SLTs were underrepresented, while service managers were not represented at all, which could have provided a useful perspective about policies as well as views about the profile and value of transcription at a whole‐service level. Future research could specifically elicit the views of adult SLTs and service managers in order to explore their perceptions.

SLTs in this study felt their transcription skills were fit for purpose; however, this study elicited self‐reported practices rather than objectively analysing SLTs’ transcription, and also did not clarify participants’ understanding of terms such as broad and narrow transcription. Future studies could analyse the actual transcription practices of SLTs in order to consider the validity of their perceptions about being fit for purpose, and to investigate the role of transcription in clinical case studies.

While some SLTs suggested CPD practices such as online packages or discussion of case studies, identifying a consensus about what CPD should include was beyond the scope of this study. This would be a valuable area to investigate in the future, and could potentially inform the development of CPD programmes, supported by some suggestions made by SLTs in this study (e.g., a focus on vowels). Similarly, establishing a consensus about service‐wide protocols for transcription by generalist SLTs (and developing this for use within services) would be a useful direction for future research, with a tangible and clinically relevant output.

### Implications for SLT

SLTs do not all use transcription in the same way, and establishing consistency across the profession is neither achievable nor, perhaps, desirable. However increased investment in good transcription practice could afford SLTs the confidence to transcribe in more detail when needed and to trust that transcriptions will be mutually understood between colleagues. While SLTs’ skills are broadly fit for purpose, this does not always apply when they encounter clients who differ from their usual populations, whose speech contains less common errors which SLTs are not always confident in perceiving and transcribing. SLTs would value and benefit from external opportunities to improve transcription skills, in order to support more reliable onward referrals and to improve diagnostic accuracy and appropriate management. Service managers could therefore promote and facilitate transcription by allowing more time when necessary, removing ‘red tape’ to enable audio‐recording and storage, investigating the use of IPA symbols in EPRs (which could also be considered by professional bodies at national level) and encouraging and funding peer support and CPD for transcription. Investing in transcription has the potential not only to promote the profession by placing value on SLTs’ unique skill, but also to inform accurate analysis of speech. This, in turn, supports appropriate management including the use of evidence‐based interventions and, ultimately, improved outcomes for clients.

## Data Availability

The data that support the findings of this study are not publicly available due to privacy or ethical restrictions.

## FOCUS GROUP QUESTIONS

In your everyday practice, what does transcription mean to you?

How often do you transcribe clients’ speech?

What sort of utterances do you transcribe?

*Prompts: Formal/informal assessments for the purposes of explicitly assessing speech, spontaneous speech that you want to capture, errors made in language assessments (e.g. a phonemic paraphasia)?*

Do you audio or video record clients, or do you always transcribe live? Or both at the same time?

*Prompts: why/why not? If so, do you set aside additional time for transcribing or for reviewing your transcription after the session? Does this help with transcribing? Why/why not? *

How do you store transcriptions?

*Prompts: handwritten into case notes / handwritten transcriptions scanned into or attached to EPR? Do you use an EPR that supports IPA?*

Are there any barriers to transcribing in everyday practice?

Does everyone in your service use transcription in the same way?

*Prompts: do you discuss this explicitly (e.g. CPD sessions)? Does your service have any guidelines for transcription best practice?*

How confident are you in transcribing

*Prompts: confidence in broad/narrow transcription? Are there any areas that you would like to improve? Has your confidence changed since qualifying? Since moving into a specialist team?*

What do you do if you are transcribing a client's speech and you're not sure about a sound that they used?

When you transcribe a client's speech, what are you really interested in capturing?

Tell me about a client whose speech you have transcribed recently.

*Prompts: did you plan to transcribe their speech before you saw them? Did you use a formal assessment tool? Was their speech appropriate or delayed/disordered? How much of their speech did you transcribe? Did you transcribe sounds, words or phrases/sentences?*

How much do you rely on transcription to support with target‐setting?

*Prompts: link to previous question – talk about whether/how the transcription informed the target‐setting. What will you do next with this client?*

What was your experience of learning transcription as a student?

*Prompts: did you have any prior experience of transcription? Did you enjoy it? How easy or difficult did you find it? How well did it relate to your clinical learning on placement?*

Do you feel able to support students to develop transcription skills on placement?

*Prompt: how do you do this?*

Do you think it's important to do this as a clinical educator?

Ability to phonetically transcribe is one of the HCPC Standards Of Proficiency. How important do you think it is in practice?

Do you have any other thoughts that you would like to share?

Can you tell me why you were interested in taking part in this group?
